# Neuroimmunomodulatory properties of polysialic acid

**DOI:** 10.1007/s10719-023-10120-z

**Published:** 2023-05-12

**Authors:** Lina Gretenkort, Hauke Thiesler, Herbert Hildebrandt

**Affiliations:** Institute of Clinical Biochemistry, Hannover Medical School, Carl-Neuberg-Straße 1, 30625 Hannover, Germany

**Keywords:** Protein glycosylation, NCAM, Neuroinflammation, Microglia, Immune checkpoint, Sialic acid-binding immunoglobulin-like lectins (Siglecs)

## Abstract

Polymeric sialic acid (polysialic acid, polySia) is a remarkable posttranslational modification of only few select proteins. The major, and most prominent polySia protein carrier is the neural cell adhesion molecule NCAM. Here, the key functions of polySia are to regulate interactions of NCAM and to balance cellular interactions in brain development and plasticity. During recent years, however, increasing evidence points towards a role of polySia in the modulation of immune responses. These immunomodulatory functions can be mediated by polySia on proteins other than NCAM, presented either on the cell surface or released into the extracellular space. This perspective review summarizes our current knowledge and addresses major open questions on polySia and polySia receptors in modulating innate immune responses in the brain.

## Microglia and neuroinflammation

Macrophages are part of the first line of defense against pathogens and engaged in tissue repair. A special type of tissue macrophages are microglia, the resident macrophage population of the central nervous system. They colonize the developing brain before the formation of the blood brain barrier, which then isolates them from the periphery [1]. Microglia therefore must fulfill functions related to immunosurveillance and tissue homeostasis that in peripheral tissues are allocated to different types of mononuclear phagocytes, i.e. macrophages and dendritic cells [DCs; 2]. In case of a disruption of the blood brain barrier, however, microglia are supported by infiltrating immune cells, including monocyte-derived macrophages that can acquire phenotypes hardly distinguishable from microglia [3, 4].

**Fig. 1:**
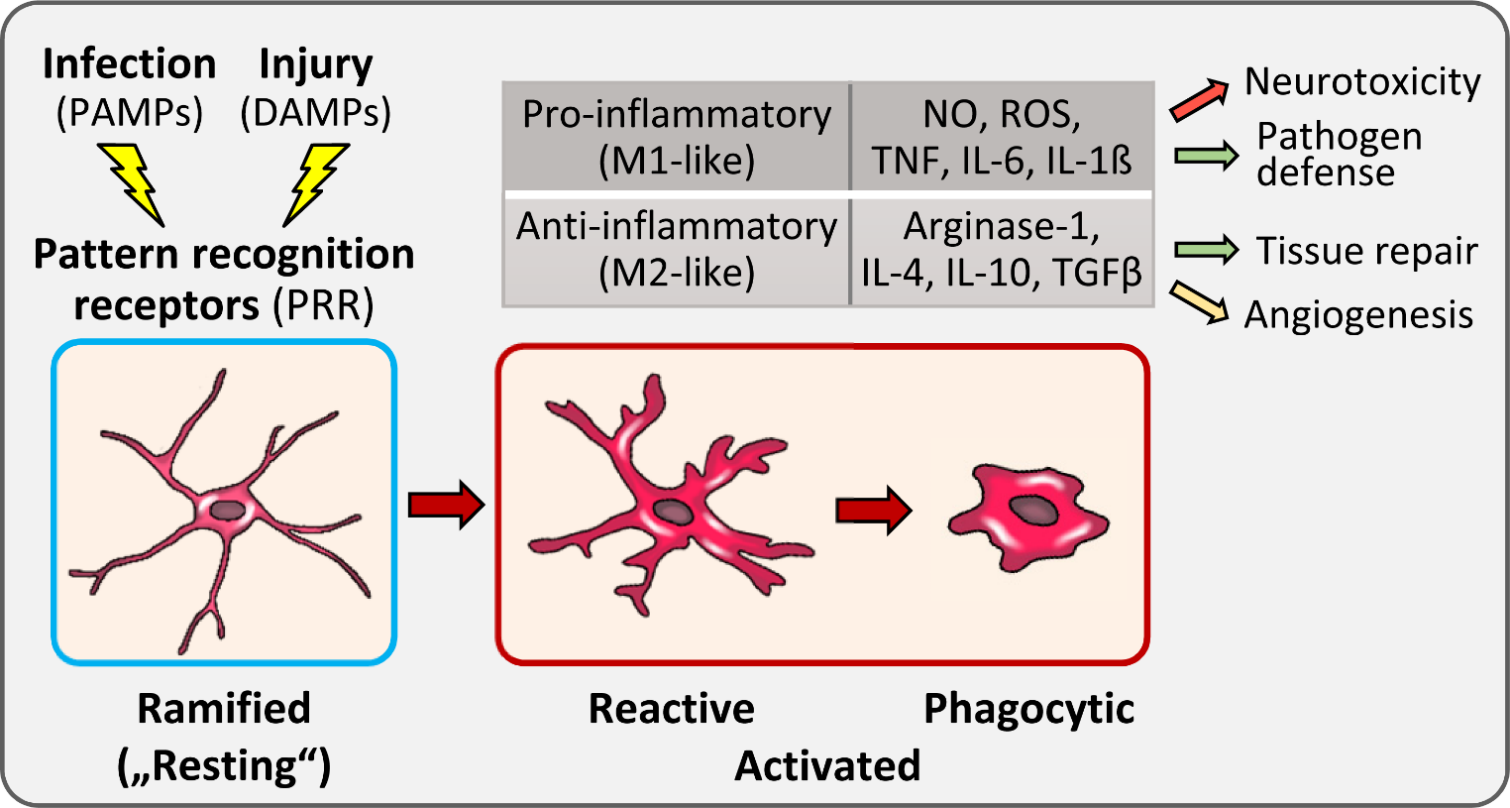
Simplified overview on microglia activation and functions. See text for details. Abbreviations: DAMPs and PAMPs, damage- and pathogen-associated molecular patterns; IL, interleukin; NO, nitric oxide; PRR, pattern recognition receptors; ROS, reactive oxygen species; TGF, transforming growth factor; TLRs, toll-like receptors; TNF, tumor necrosis factor

Depending on brain region and species, numbers of microglia vary greatly and estimates range from 5 to 12% of all cells in the brain parenchyma of mice and from 0.5 up to 17% in the human brain [5, 6]. The morphological appearance of microglia depends on their activation status. In the homeostatic, non-activated state microglia display highly branched cellular processes and are also referred to as ramified (Fig. 1). Previously designated “resting” or “quiescent”, intravital imaging revealed that these microglia show constant motility of their cellular processes within the CNS tissue in order to survey their environment in a non-overlapping territorial fashion [7, 8]. In response to infection or tissue damage by traumatic brain injury or other insults, pathogen- or damage-associated molecular patterns (PAMPs, DAMPs) are sensed by pattern recognition receptors leading to microglia activation [9, 10]. This causes a dramatic change in microglia morphology, involving a rapid reorientation of processes towards the site of injury, followed by a reactive state, characterized by a shortening and thickening of cellular processes, and finally an amoeboid shape with full phagocytic capacity (Fig. 1). Similar to other tissue macrophages, microglia can polarize towards functionally different activation states that are broadly categorized into classical inflammatory (M1-like) and alternative anti-inflammatory activation (M2-like) [11, 12].

Inflammatory activation is mediated by pattern recognition receptors like the toll-like receptors (TLRs), for example the endotoxin receptor TLR4, sensing bacterial lipopolysaccharides (LPS). This induces the production of toxic nitric oxide (NO) and reactive oxygen species (ROS) to neutralize pathogens, as well as expression and release of proinflammatory cytokines, such as tumor necrosis factor (TNF), interleukin (IL)-6, and IL-1ß to promote local inflammatory activation, and of a number of chemokines to attract additional immune cells. Hallmarks of M2-like activation are the expression of arginase-1 antagonizing NO synthesis, and the production of anti-inflammatory cytokines such as IL-4, IL-10 and IL-13 (Fig. 1). However, especially for microglia, the classification of macrophage activation into M1- and M2-like states cannot adequately reflect the complex situation in vivo [13]. Nevertheless, particularly for in vitro studies, it still is a helpful concept for the characterization of microglia activation states.

Balancing pro- and anti-inflammatory activation in the damaged brain is crucial for efficient repair while preventing overshooting immune responses [14]. Neuroinflammation may be beneficial or detrimental depending on the context (Fig. 1). It is indispensable for pathogen defense but also implicated in neurotoxicity and neurodegenerative diseases [11, 15, 16]. Along the same lines, anti-inflammatory states are related to tissue repair and angiogenesis, but in the context of neovascularization in brain tumor development the latter can be fatal [17]. More general, the unique, but overall anti-inflammatory profile of microglia and macrophages associated with brain tumors such as gliomas favors tumor progression and is linked to poor prognosis [18]. Together, this highlights that understanding the mechanisms that control microglia activation and recruitment is essential for therapeutic interference with inflammation in brain disease.

## Polysialic acid: Brief synopsis on structure, biosynthesis, cell surface presentation, and NCAM modulation

Polysialic acid (polySia) is the generic term for linear polymers of sialic acids (a.k.a. neuraminic acids) with variable lengths, i.e. degrees of polymerization (DP). In vertebrates, polySia consists of at least eight and up to 90 or more *N*-acetylneuraminic acid residues linked by α2,8 glycosidic bonds [19, 20]. As a unique posttranslational modification of only a small number of select proteins, polySia can be added to terminal sialic acids on *N*- and *O*-glycans by two different enzymes of the trans-Golgi compartment, the polysialyltransferases ST8SIA2 and ST8SIA4, which show distinct but often overlapping expression patterns [21–23]. Notably, polySia with the exact same structure but a completely unrelated biosynthetic pathway is found as a capsular polysaccharide called colominic acid on certain pathogenic bacteria [24, 25]. PolySia is mostly presented on the cell surface and alters basic biophysical properties of its protein carrier. Thereby, polySia regulates specific protein functions, but also is able to modulate overall cell surface interactions with major implications for nervous system development and plasticity [19, 26]. In this regard, the by far most abundant and best studied carrier presenting polySia at the surface of neural lineage cells, namely oligodendroglia, astroglia and neurons, is the neural cell adhesion molecule NCAM, which can be polysialylated by each of the two polysialyltranferases individually or combined [27]. Numerous comprehensive reviews highlight the divers roles of polySia-NCAM and the impact of its dysregulation on mostly mouse brain development, structural and synaptic plasticity, or nervous system repair, with consequences for learning, memory, and cognition, as well as for setting a neurodevelopmental predisposition to psychiatric diseases [see for example 19, 22, 25, 28, 29, 30].

## Polysialic acid of neural lineage cells on proteins other than NCAM

The remarkably specific polysialylation of NCAM is highlighted by the observation that the brain of NCAM-negative mice is almost, but not completely, devoid of polySia [31]. Indeed, a few other carriers of polysialic acid have been identified and some of them were also detected in the brain [21]. Occurrence of polySia has been reported on sodium channel alpha subunits in synaptosomal fractions of adult rat brain [32] and on SynCAM 1 (gene name *Cadm1*) at a few, rather undefined sites during perinatal development of the mouse brain [33]. In both cases, the physiological consequences of the polySia modification remain elusive. Analyses of a muscular sodium channel alpha subunit expressed in CHO cells capable or not capable of polysialylation indicated that polySia has a stabilizing effect on voltage-dependent gating [34]. Concerning SynCAM 1, the most prominent function is its ability to induce synapse formation of neurons [35]. In contrast, polysialylated SynCAM 1 was not detected in neurons, but associated with NG2-positive oligodendrocyte precursor cells (OPCs) of murine and human origin [33, 36, 37]. This cell population mainly gives rise to myelinating oligodendrocytes in development, maintenance and repair of myelin, the insulating sheath around axons that allows rapid nerve conduction and also provides metabolic support to maintain axonal integrity [38]. OPCs can receive transient synaptic input from neurons prior to further differentiation [39–41], and recent data from zebrafish suggest that a subpopulation of OPCs integrates neuronal information and proliferates to generate another OPC subpopulation that executes myelin formation [42].

Despite this progress, the relevance of neuron-OPC synapses remains poorly understood [43, 44]. However, the dynamic regulation of neuronal synapse formation and maintenance by SynCAM 1 [45], together with the abrogation of its adhesive properties by polySia [33], raised the possibility that the generation of polySia-SynCAM 1 by OPCs may play a role in the turnover of neuron-OPC synapses. With regard to this possible function of polySia-SynCAM 1, it was intriguing that about 20% of mouse OPCs in vitro were negative for NCAM and positive for polySia-SynCAM 1, which accumulated in the Golgi compartment, but was transiently recruited to the cell surface in response to depolarization [37]. Yet, a physiological function could not be assigned to polySia-SynCAM 1. In vivo and in vitro, polySia on SynCAM 1 is exclusively produced by ST8SIA2 [37, 46] and *St8sia2*-negative mice show deficits of developmental myelination and myelin maintenance, which may be caused by impaired transition from OPCs to myelinating oligodendrocytes [47]. A potential contribution of polySia-SynCAM 1 was not investigated in this study, but in a mouse model of cuprizone-induced de- and remyelination, the deficits of myelin repair and OPC differentiation in *St8sia2*-negative mice were faithfully reproduced by *Ncam* deficiency, and even during the phase of highest OPC recruitment no polySia-SynCAM 1 could be detected [48]. Thus, a prominent role of polySia-SynCAM 1 in myelination and myelin repair appears unlikely. In contrast, increasing evidence points towards roles of OPCs beyond myelination, such as a modulation of neuroinflammation [44, 49]. Therefore, polySia-SynCAM 1 produced by OPCs may contribute to the immunomodulatory functions of polySia as discussed in the following sections.

## Polysialic acid in microglia and macrophages

**Fig. 2:**
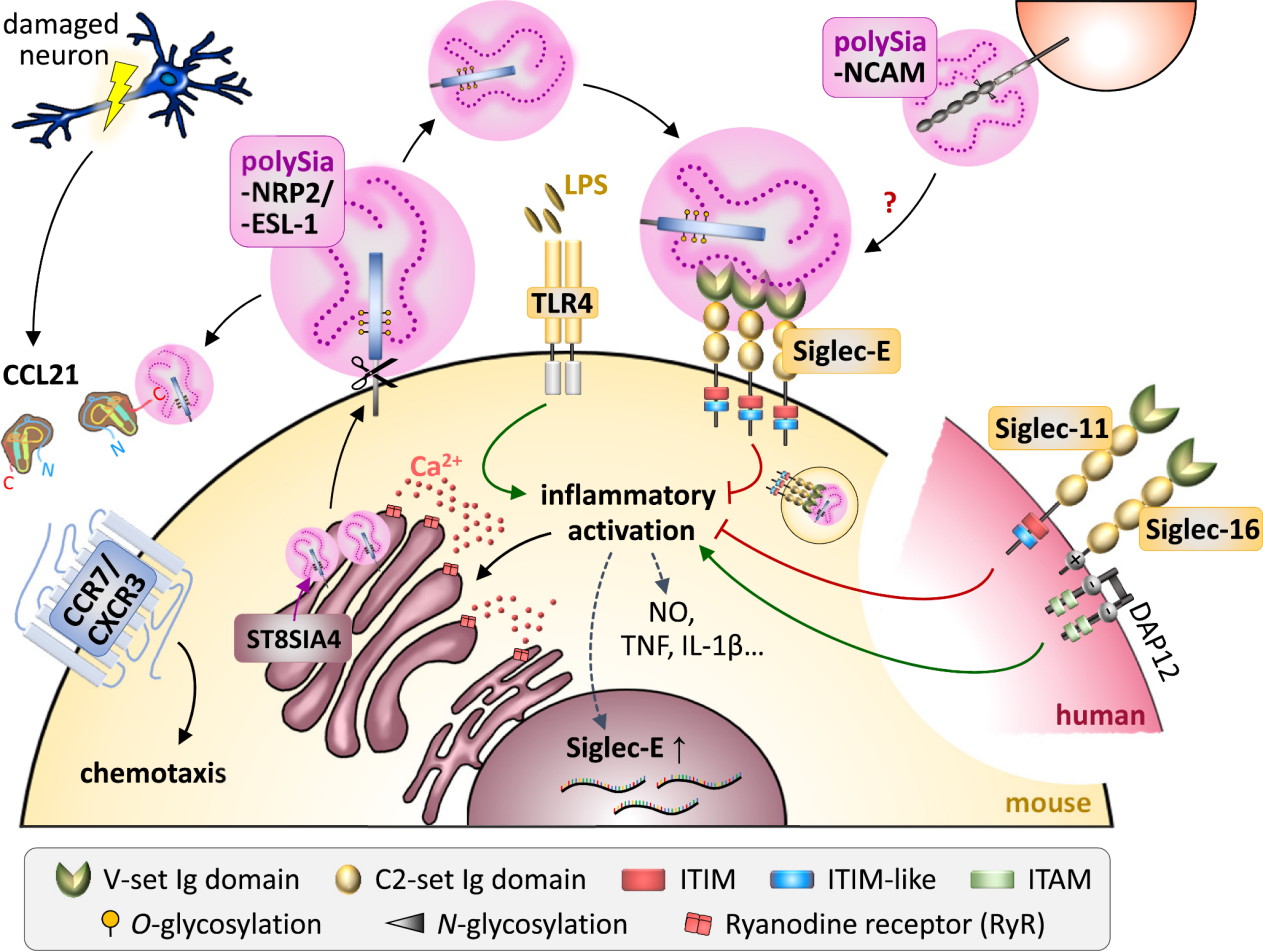
Working model of the proposed impact of polySia and polysialylated proteins on microglia activation and recruitment. See text for details. Abbreviations: CCL, C-C motif ligand; CCR, C-C motif chemokine receptor; CXCR C-X-C motif chemokine receptor; DAP12, DNAX-activating protein of 12 kDa; Ig, immunoglobulin; ITAM, immunoreceptor tyrosine-based activation motif; ITIM, immunoreceptor tyrosine-based inhibitory motif; Siglec, sialic acid-binding immunoglobulin-like lectin. Others: see legend to Fig. 1

Strikingly similar to the Golgi-enrichment and activity-dependent recruitment of polySia-SynCAM 1 in OPCs, cultured murine microglia and human THP1 macrophages accumulate polysialylated proteins in the Golgi compartment, and, in response to activation, which in this case is induced by inflammatory LPS treatment, these polysialylated proteins are translocated to the cell surface and released [37, 50] (Fig. 2).

One of these polysialylated proteins is neuropilin-2 (NRP2), well-known as a regulator of axon guidance and angiogenesis [51]. NRP2 has first been identified as a polySia carrier on the surface of human monocyte-derived DCs [52]. In DCs, cell surface polysialylation increases with maturation and a first functional analysis indicated an inhibitory role of polySia-NRP2 in T cell activation and proliferation mediated by mature DCs [52]. As further detailed in the section on “PolySia-chemokine interactions”, polySia on mature DCs is also indispensable for their chemotactic migration towards CCL21. Based on the knowledge on polySia-NRP2 expression by DCs, the presence of polySia-NRP2 in microglia was detected by analyzing polySia immunoreactivity of primary microglia cultures derived from the early postnatal brain of NCAM knockout mice [37].

The second polysialylated protein in microglia and macrophages is E-selectin ligand-1 (ESL-1; gene name *Glg1*). As the name implies, ESL-1 is a cell adhesion ligand of E-selectin, implicated in leukocyte rolling [53, 54]. It was first described as Golgi-localized sialoglycoprotein MG-160 [55] regulating TGF-ß maturation during cartilage and bone homeostasis [56, 57], and as a cysteine-rich fibroblast growth factor receptor [58] implicated in FGF and heparin binding at the cell surface [59]. ESL-1 was selected as a candidate polySia carrier from a list of proteins identified by mass spectrometry in lysates from two different mouse embryonic stem cell (ES)-derived microglia lines that have been enriched for polysialylated proteins by either immunoprecipitation (IP) with the polySia-specific monoclonal antibody 735 or affinity precipitation with an inactivated phage-derived endosialidase [50]. Subsequently, polysialylation of NRP2 and ESL-1 was unequivocally demonstrated by Western blot analyses after IP with polySia-specific antibodies and, reversely, after IP with NRP2- or ESL-1-specific antibodies. The simultaneous occurrence of polySia-NRP2 and polySia-ESL-1 could be confirmed for the two ES-derived microglia lines used in the glycoproteomic approach, for primary cultured mouse microglia and human THP1 macrophages as well as for injury-induced microglia in brain slice cultures from wildtype and *Ncam* knockout mice [50] and, in a later study, also for the murine microglial cell line BV2 [60].

In contrast to polySia on NCAM or SynCAM 1, which is attached to complex *N*-glycans by either both polysialyltransferases or specifically by ST8SIA2 (see above), polysialylation of NRP2 was found to be performed only by ST8SIA4 and occurs exclusively on mucin-type *O*-glycans [61]. The glycosylation sites that harbor polySia on ESL-1 have not been analyzed yet. Evidently, however, ESL-1 is also polysialylated by ST8SIA4 on *O*-glycans, because the Golgi-resident polySia was (i) still present in microglia derived from *Ncam* and *St8sia2* double-knockout mice, but completely absent from NCAM- and ST8SIA4-negative microglia and (ii) unaffected by enzymatic digestion of *N*-glycans with PNGaseF but eliminated by inhibition of *O*-glycan synthesis with benzyl-2-acetamido-2-deoxy-α-D-galactopyranoside (benzyl-α-GalNAc) [37].

The prevailing model of polySia as a modulator of cell surface interactions seems hardly compatible with the Golgi-localization of polysialylated proteins in microglia. However, upon inflammatory activation of microglia with LPS, the Golgi-confined pool of polySia was rapidly translocated to the cell surface [37, 50]. By immunostaining of fixed, non-permeabilized ES-derived microglia, polySia at the cell surface could be detected between ten minutes and about one hour after LPS induction, before the signals disappeared, so that LPS-induced cells with the amoeboid morphology of activated microglia became negative for polySia on the cell surface and in the Golgi. In contrast, the Golgi-like polySia pattern remained unaltered and no cell surface translocation was observed, when the cells were activated by IL-4 to induce microglia with an amoeboid morphology, but an M2-like profile characterized by arginase 1 immunostaining [37].

Pretreatment with the anti-inflammatory drug minocycline inhibited the appearance of amoeboid cells and reduced the LPS-induced loss of Golgi-resident polySia [37]. Furthermore, inhibition of metalloproteinases prevented the polySia depletion in response to LPS treatment and polySia signals were maintained on the surface of cells with the morphology of activated microglia [50]. Together, this indicates that inflammatory activation induces metalloproteinase-mediated ectodomain shedding of polysialylated proteins. Indeed, by polySia-IP and Western blot analyses polySia-NRP2 and polySia-ESL-1 could be detected in cell culture supernatants of LPS-induced microglia. A comparison of the protein bands after enzymatic removal of polySia with endosialidase revealed that NRP2 and ESL-1 immunoreactive bands appeared at a lower apparent molecular weight as compared to the respective bands obtained from cell pellets, indicating a smaller protein backbone of the polySia-NRP2 and polySia-ESL-1 species in the supernatant [50]. This supports that the polysialylated proteins are released from the cell surface by ectodomain shedding (indicated by the scissors symbol in Fig. 2).

In ES-derived murine microglia and human THP1 macrophages, polySia was detected on only small fractions of NRP2 and ESL-1, and out of several NRP2 isoforms, only one with an apparent molecular weight of about 125 kDa was polysialylated [50]. In the case of ESL-1, polySia was associated with a band at about 150 kDa. This was the only ESL-1 species found in murine microglia, but for human THP1 macrophages, Western blot analysis revealed a second ESL-1 band at a higher molecular weight that was not polysialylated and, based on its electrophoretic migration, appeared too big to represent a second isoform reported for human ESL-1 [50]. Before and after LPS treatment, NRP2 immunoreactivity was distributed over the entire cell, while ESL-1 signals were always confined to the Golgi compartment and both did not parallel the LPS-induced loss of polySia.

The trigger for the release of the polysialylated proteins from the Golgi compartment in response to the inflammatory activation of microglia seems to be a depletion of calcium (Fig. 2). The mobilization of calcium from intracellular stores is a central element of LPS-induced microglia activation and involves the activation of ryanodine receptors (RyRs) [9, 62]. The trans-Golgi compartment, where polysialylation takes place, is also a calcium store that can be selectively mobilized by activation of RyRs [63, 64]. Remarkably, in the absence of LPS, a translocation of polysialylated proteins from the Golgi compartment to the cell surface, but not their release, could be induced by treatment with an RyR agonist, whereas an RyR antagonist was able to prevent the LPS-induced release [60]. Hence, because NRP2 and ESL-1 share no obvious feature other than polysialylation, calcium-dependent changes of polySia interactions may underlie the common regulation of Golgi retention and LPS-induced mobilization of polySia-NRP2 and polySia-ESL-1 from the Golgi compartment. During polysialylation, ST8SIA4 interacts with nascent polySia chains and with the protein acceptor [65–67]. As shown for polySia antibody binding, calcium affects polySia-protein interactions, probably by stabilizing conformational states of polySia epitopes [19, 68]. In this way, calcium-dependent interactions between polySia and ST8SIA4 could mediate retention of polySia-NRP2 and polySia-ESL-1 in the Golgi compartment, and calcium depletion may trigger their release.

Analyses by immunostaining indicated a rapid discharge of polysialylated proteins within the first hour after LPS-induction of microglia [37, 50]. In contrast, monitoring by immunoaffinity chromatography revealed that cultured microglia continuously release protein-bound polySia for at least 24 h after LPS induction [60]. This implies that the amount of d polySia can be much higher than anticipated based on immunostaining and that analysis by immunostaining will not be suited to detect a potential release of polysialylated proteins by activated microglia in, e.g., chronic states of neuroinflammation.

Evidently, primary cultured microglia experience activation during isolation and cultivation which is problematic to define and can hardly be matched to a particular activation state in vivo [69, 70]. The same applies to cell lines cultured in the presence of serum, and, a little less though, to ES-derived microglia derived under serum-free conditions [71]. It therefore is crucial to validate findings obtained in vitro by observations in situ or in vivo. Concerning the expression of polySia by cultured microglia the data are conflicting. Polysia-NCAM was detected on the cell surface of primary cultured microglia obtained from early postnatal mouse brain [37, 72] as well as on the mouse microglial cell line Ra2 [73]. In contrast, analyses of two different mouse ES-derived microglia lines and of the widely used microglial cell line BV2 by immunostaining, IP and Western blot yielded no NCAM, polySia-NCAM, or cell surface polySia staining, and NRP2 and ESL-1 were the only detectable carriers of polySia [50, 60].

With regard to these discrepancies the following observations are worth mentioning: First, primary cultured microglia express markers unique to peripheral monocytes indicating a deviating phenotype in vitro [70]. Second, human THP1 cells show a remarkable switch from the expression of polySia-NCAM on the cell surface at the monocyte-like stage towards the expression of Golgi-localized polySia on NRP2 and ESL-1 after phorbol ester-induced differentiation into a macrophage-like state [50]. Third, despite large amounts of polySia on neurons, microglia in organotypic slice cultures from early postnatal mouse brain were devoid of polySia at the cell surface, but between one and five days of cultivation, an increasing amount of the injury-induced microglia displayed Golgi-localized polySia signals that could be abolished by LPS treatment [50]. Due to the downregulation of polySia during postnatal mouse brain development [74], brain slice cultures from older mice were largely devoid of polySia-NCAM on neurons. Hence, in many parts of these slice cultures, the Golgi-like polySia signals of activated microglia were the only source of polySia immunoreactivity [50]. Taken together, it seems obvious that microglia under physiological conditions are devoid of polySia-NCAM and produce polySia on NRP2 and ESL-1 upon activation by injury or DAMPs present in, e.g., serum-supplemented cell cultures.

**Fig. 3:**
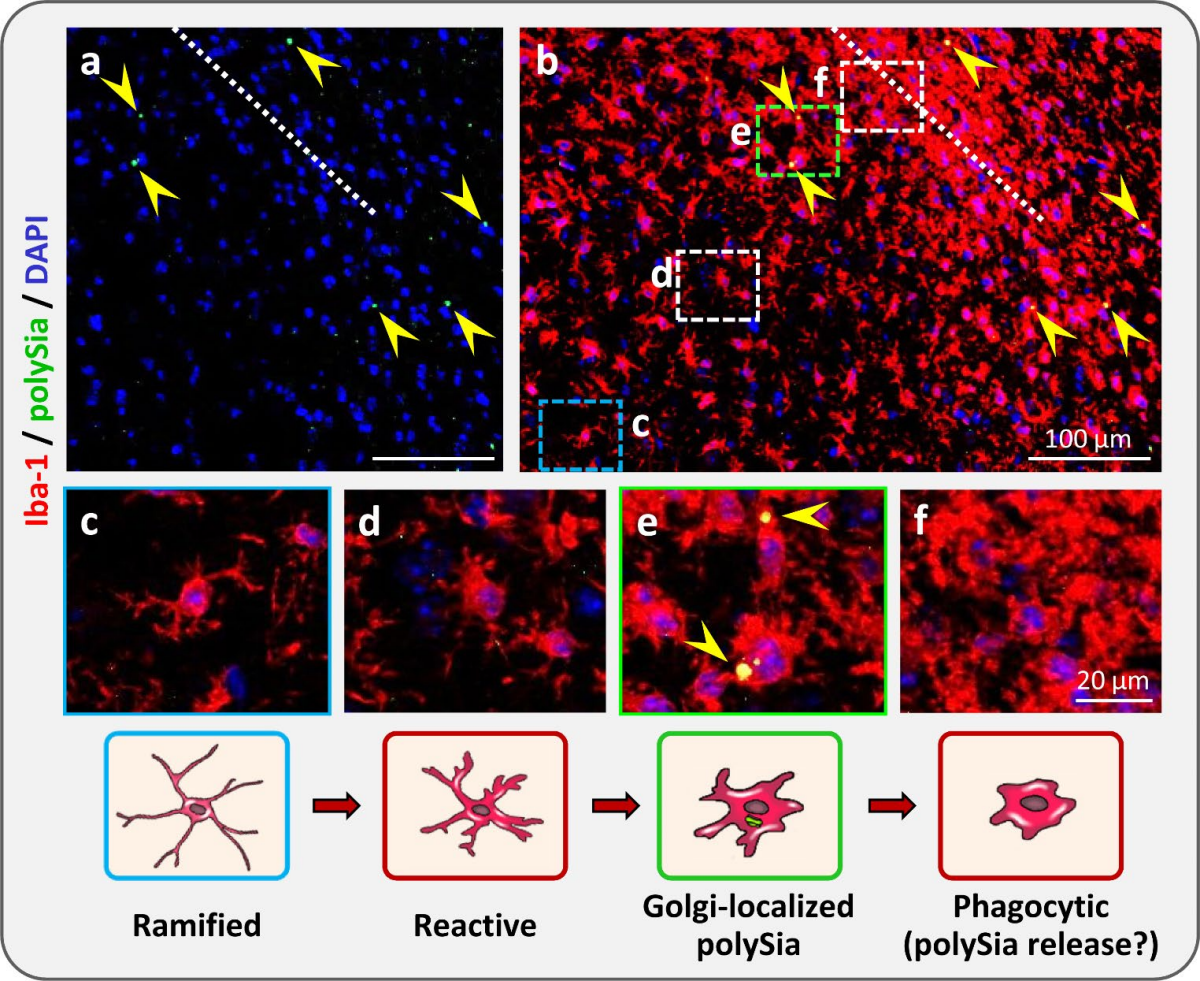
PolySia in microglia one week after activation by traumatic brain injury (TBI). **a-b**, Immunofluorescence staining of polySia (green, yellow arrowheads) combined with a nuclear counterstain (DAPI, blue) in (a) and, additionally, with the microglia marker Iba-1 (red) in (b). The dotted line indicates the position of the wound channel. **c-f**, Higher magnifications of the boxed areas in (b) and corresponding schemes of microglia with morphologies indicative for different states of activation, as described in the text. Modified from [60]

Proof of principle that polySia can be produced by activated microglia in vivo was obtained in a mouse model of traumatic brain injury (TBI) [60]. Seven days after a small confined stab lesion to the cortex, numerous cells positive for the microglial marker Iba-1 and featuring the morphology of activated microglia could be detected around the wound channel (Fig. 3). Some, but only few of these cells displayed the characteristic Golgi-like polySia staining previously detected in vitro and in slice cultures. They were all located in some distance to the wound channel, suggesting that microglia in close proximity to the lesion site lost polySia immunoreactivity upon further activation. In analogy to the continuous shedding of polysialylated proteins by activated microglia in vitro, this points towards a release of polysialylated proteins by injury-activated microglia in vivo. With increasing distance from the wound, reactive microglia with short, thick processes appeared and finally displayed ramified morphologies, indicative for the homeostatic state. In both of the latter states, microglia were negative for polySia. Based on these findings, it can be assumed that cultured microglia, which consistently display accumulations of polySia in the Golgi compartment, correspond to a transient injury-induced activation state of polySia-positive microglia in TBI.

## Immunomodulation by the polySia-Siglec axis: In vitro evidence, conjectures, and pending questions

The first study linking polySia to microglia activation described that polySia-NCAM on neurons was able to reduce neurotoxicity of murine microglia that has been transduced with Siglec-11 [72]. Siglecs (sialic acid-binding immunoglobulin-like lectins) are among the receptors that are able to modify the activity of brain resident microglia and of immune cells infiltrating the damaged brain [75]. They are ‘immuno globulin-type’ (I-type) lectins that bind sialylated glycans with an amino-terminal V-set immunoglobulin (Ig) domain [76–79]. Conventionally, Siglecs are divided into those that are structurally conserved across mammals and the group of CD33 (Siglec-3)-related Siglecs that vary considerably between species. In contrast to ten functional CD33-related Siglecs in humans, only five members of this group are known in mice [77, 79]. In their cytoplasmic domain, most CD33-related Siglecs have immunoreceptor tyrosine-based inhibitory motifs (ITIMs), which signal through tyrosine phosphorylation by Src-family kinases and recruitment of mainly the protein tyrosine phosphatases SHP-1 and SHP-2. The ITIM-containing receptors counteract signaling from receptors associated with an immunoreceptor tyrosine-based activation motif (ITAM), such as the triggering receptor expressed on myeloid cells 2 (TREM2) [76–79].

Siglec-11 is an ITIM-containing human immune receptor of microglia and other tissue macrophages that has no direct counterpart in mice [80, 81]. However, in search for possible functions of the polysialylated proteins released by murine microglia, it became apparent that soluble polySia efficiently inhibits the LPS-induced activation of murine primary cultured and ES-derived microglia, indicating the presence of an inhibitory polySia receptor in mice [37, 50].

**Fig. 4:**
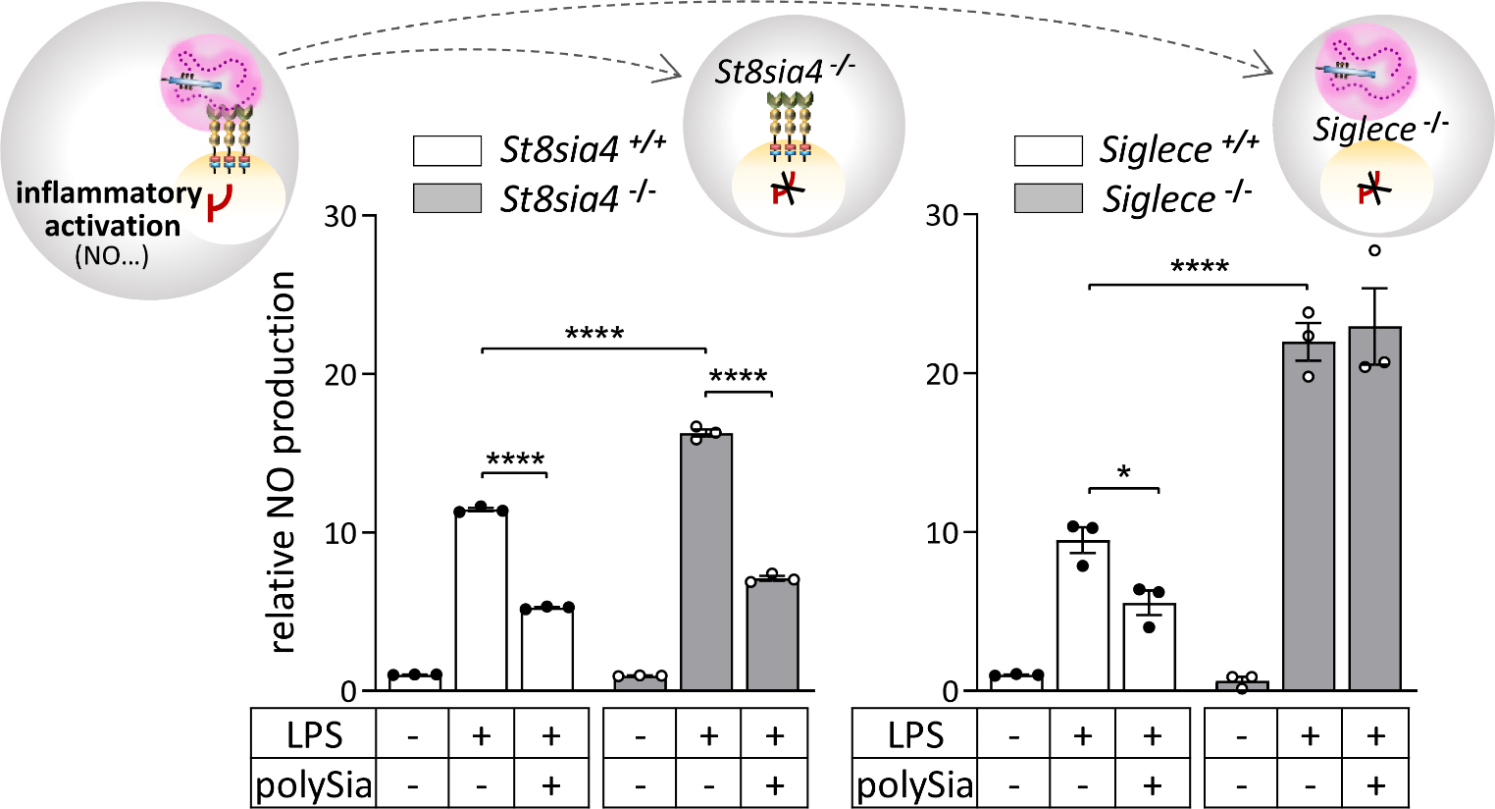
Loss of ST8SIA4 and Siglec-E potentiates the microglial response to LPS. NO production in response to LPS is significantly increased in primary microglia derived from NCAM- and ST8SIA4-negative mice (*St8sia4*^−/−^) as compared to NCAM-negative mice with wildtype *St8sia4* (*St8sia4*^+/+^, left panel), as well as in Siglec-E-deficient (*Siglece*^−/−^) as compared to wildtype BV2 microglia (*Siglece*^+/+^). Consistent with the inability to produce polySia as a ligand of Siglec-E, or the loss of Siglec-E as a receptor of polySia, addition of free, soluble polySia to LPS-induced cells is still able to inhibit ST8SIA4- but not Siglec-E-deficient microglia. For each graph, data were normalized to untreated controls, and, after two-way ANOVA, results of Holms–Sidak’s post hoc tests are shown for selected group comparisons (* *p* < 0.05, **** *p* < 0.0001). Data compiled from Werneburg et al. [37], with permission from Wiley, and from Thiesler et al. [60]

Dampening inflammatory activation of microglia by free, soluble polySia implicated a negative feedback mechanism through the release of protein-bound polySia. This was corroborated by the finding that primary cultured microglia from NCAM and ST8SIA4 negative mice, i.e. microglia that is no longer able to release polysialylated NRP2 and ESL-1, show a significantly higher activation in response to LPS, when compared to microglia with wildtype ST8SIA4 obtained from mice lacking NCAM and ST8SIA2 [37] (Fig. 4, left).

Searching for the murine polySia receptor, Siglec-E appeared to be a promising candidate, because Siglec-E is a major inhibitory Siglec of murine microglia and macrophages [82, 83] and in vitro data indicated that polySia encapsulated *E. coli* K1 bind to the extracellular part of Siglec-E as efficiently as to human Siglec-11 [84]. However, Siglec-E is considered to be a promiscuous receptor and in a glycan array study, Siglec-E-Fc chimeric protein bound to a wide range of sialoglycans [85]. Among them were α2,8-linked di- and trisialic acid (DP2, DP3), showing a manifold higher Siglec-E binding than oligo- and polysialic acids with DP4, 6, 8, 10 or 11. Likewise, studies with a Siglec-11-Fc chimera indicated binding of short oligomers [80]. In contrast, LPS-induced inflammatory activation of human THP1 macrophages could be attenuated by experimentally added soluble polySia with an average DP of 20 (avDP20), but not by oligosialic acid with DP6, and lentiviral knockdown of Siglec-11 eliminated the inhibitory effect [86]. Similarly, knockdown of Siglec-E from murine ES-derived microglia abolished the anti-inflammatory impact of avDP20 [87], but, as emphasized by the authors, in mouse microglia with uncompromised Siglec-E expression, about tenfold more avDP20 (1.3µM) was needed to elicit the same inhibitory response as for the Siglec-11-mediated inhibition of human THP1 macrophages (140nM). Contradictory at first sight, 30 nM of polySia with chain lengths ranging from DP5 to DP > 100 and an average DP of 50 (avDP50) was sufficient to inhibit the LPS-induced activation of NO production in murine BV-2 microglia and CRISPR/Cas9 mediated knockout of Siglec-E in these cells prevented inhibition, even when tenfold more (300nM) polySia was applied [60]. The data therefore infer that the DP of polySia needed for optimal activation of the inhibitory receptors differ between Siglec-E and Siglec-11 in a way that the polySia preparation with avDP20 used by Karlstetter et al. [87] efficiently interacts with Siglec-11, but contains only a minor fraction of polySia with a chain length able to activate Siglec-E signaling.

Besides the loss of inhibition by experimentally added polySia, Siglec-E deficiency resulted in a strong increase of the LPS response [60] that was comparable to the higher responsiveness of primary cultured microglia with abolished polySia synthesis (Fig. 4). Together, these data clearly argue that the release of polysialylated proteins by activated microglia is part of a negative feedback mechanism, in which polySia acts as a trans-activating ligand of Siglec-E to prevent an overshooting neuroinflammatory response.

The underlying mechanisms responsible for the marked differences between the binding properties of Siglec-E in glycan arrays compared to the Siglec-E mediated anti-inflammatory activity, as well as for the divergent responses of Siglec-E and Siglec-11 to different polySia preparations remain to be explored. Of note, clustering of both Siglec receptors and their ligands seems essential for high-avidity interactions [76]. Accordingly, the multivalent display of disialic acids (DP2) on nanoparticles, but not free soluble DP2, was able to enhance the oligomerization of Siglec-E and to inhibit inflammatory activation of macrophages [88]. This raises the possibility that polySia, in contrast to sialic acid oligomers, is able to induce inhibitory signaling by presenting multiple interaction motifs to organize receptor clustering (as indicated in Fig. 2). In keeping with this assumption, the minimal chain lengths needed for multivalent binding may differ between Siglec-E and Siglec-11. Such an interaction would either require that moieties within the polySia chain form interacting motifs, also designated *endo* binding mode [89], as described for binding sites of polySia-specific antibodies [90, 91] or the endosialidase from the bacteriophage K1F (endo NF) [92, 93]. Alternatively, if only the nonreducing end of polySia engaged in interactions with the conserved sialic acid-binding site of the V-set Ig domain in an *exo* binding mode, as shown for the interaction of polySia with the short fiber knob of the human adenovirus 52 [89, 94], clustering would require additional polySia binding sites of the corresponding Siglec.

With regard to the expression of Siglec-E in response to inflammatory activation, a strong increase of Siglec-E on the mRNA level contrasted with immunostaining results showing a drastic downregulation of Siglec-E cell surface presentation after LPS induction of BV2 microglia [60]. A similar reduction of Siglec-E at the cell surface could be induced by application of free, soluble polySia to otherwise untreated cells, whereas pharmacological inhibition of clathrin-independent endocytosis prevented the downregulation and caused a massive accumulation of Siglec-E at the surface of LPS-induced microglia. These findings suggest an internalization of Siglec-E in response to interaction with polySia, further supporting that Siglec-E acts as polySia receptor (Fig. 2). Indeed, co-localization of polySia and Siglec-E with the endosomal marker early endosomal antigen 1 (EEA1) could be detected after LPS-induction [60].

Endocytosis of Siglecs by clathrin-dependent or -independent mechanisms has been observed in response to antibody ligation or interactions with multivalent ligands, but the consequences of the receptor internalization are not clear [78, 95, 96]. Likewise, it remains open how the endocytosis of polySia and Siglec-E may contribute to Siglec-E mediated inhibitory signaling and it is not yet known if human Siglec-11 undergoes a similar internalization. Nevertheless, it appears likely that the uptake of the Siglec receptor together with its ligand limits the availability of soluble polySia or polysialylated proteins. Both, signaling and ligand depletion may be different for intercellular signaling interactions of Siglecs with polySia presented by proteins on the surface of neighboring cells, such as polySia-NCAM on neurons (as indicated in Fig. 2), because this configuration may not allow for an internalization of the polySia ligand together with an interacting Siglec receptor. However, it should be noted that after downregulation of polysialylation during postnatal brain development [74], polySia-NCAM is actually rare in most parts of the healthy adult brain [19, 97]. In contrast, the re-expression of polySia-NCAM is a hallmark of many tumors, including brain tumors, such as medulloblastoma and gliomas, including glioblastoma [27, 98–102]. Up to now, however, nothing is known about the potential impact of polySia-NCAM interactions with Siglecs of the tumor immune environment^1^.

In the frame of a study on the engineering of protein sialylation in plants, it could be demonstrated that protein-bound polySia, in this case attached to an NCAM fragment, is as potent as free soluble polySia in reducing the LPS-induced production of nitric oxide by BV2 microglia [103], indicating that this immunomodulatory function of polySia is independent from its underlying protein carrier. It therefore is puzzling and so far unresolved, why microglia release two different polysialylated proteins in response to LPS. The two polySia carriers NRP2 and ESL-1 may differ in their interactions with ST8SIA4, which could affect rates of polySia synthesis and eventually result in different lengths of the resulting polySia chains. Differences between the two proteins may also be relevant for the regulation of ectodomain shedding as well as for the availability and accessibility of polySia in the extracellular environment. For example, ESL-1 interacts with heparan sulfate proteoglycans [59] and therefore binds to the extracellular matrix, which may be important to limit diffusion and increase the local concentration of polySia-ESL-1 after shedding. Furthermore, as for polySia on NCAM and SynCAM 1 (see above), it can be assumed that polysialylation of NRP2 and ESL-1 also modulates protein-specific functions either in regulating interactions within the Golgi compartment, during the short period of cell surface presentation, or after shedding.

Since immunomodulation by the polySia-Siglec axis seems independent from functions of the polySia protein carriers, release of polySia-SynCAM 1 by OPCs may add to a pool of polySia implicated in modifying neuroinflammation in vivo. OPCs are abundant throughout the adult brain, highly mitotically active and, based on single-cell RNA sequencing datasets, comprise numerous subtypes, while only a small percentage is needed for myelin maintenance [44]. Because OPCs are also highly sensitive to proinflammatory cytokines and, in response, express a number of cytokines, chemokines and other immunomodulatory factors themselves, it has been suggested that OPCs and OPC crosstalk with microglia contribute to the regulation of neuroinflammation [44, 49]. Concerning polySia-SynCAM 1, the experimental evidence is limited to the observation that, in the subset of polySia-NCAM negative cells in primary mouse OPC cultures, depolarization leads to translocation of polySia-SynCAM 1 from the Golgi compartment to the cell surface before the cells loose polySia immunoreactivity [37]. However, not only this sequence, but also the time window of about one hour between the depolarizing treatment and the loss of cell-associated polySia is highly reminiscent to the findings in microglia. Also similar to the inflammatory activation of microglia, depolarization of OPCs leads to a calcium release from internal stores that involves the activation of RyRs [104].

So far, it is not known if a release of polysia-SynCAM 1 from OPCs can be caused by neuroinflammatory signals. Yet, protein ectodomain shedding by cells of the oligodendrocyte lineage, including OPCs, contributes significantly to the secretome of the adult mouse brain [105], and all polysialylated isoforms of SynCAM 1 contain the variably spliced exon 8b [46] coding for 11 amino acid residues that are necessary and sufficient for SynCAM 1 ectodomain shedding [106]. Considering, furthermore, that the continuous release of polysialylated proteins by activated microglia was undetectable by immunostaining [60], it seems possible that ectodomain shedding of polySia-SynCAM 1 by OPCs takes part in the control of neuroinflammation, although polySia-SynCAM 1 could not be detected during the inflammatory response to cuprizone-induced demyelination [48]. In this regard, since polySia on SynCAM 1 is produced by ST8SIA2, it is interesting that a recent study reports a link between the mRNA expression level of ST8SIA2, regulated by a network of circular RNA and microRNA interactions, and immune regulation in a mouse model of postoperative neurocognitive disorder [107].

## Siglec-11 and Siglec-16 as paired polySia receptors of human microglia and macrophages

Experimentally added soluble polySia with avDP20 has been demonstrated to interact with Siglec-11 and to attenuate LPS-induced inflammatory activation of human THP1 macrophages [86]. Interestingly, expression of Siglec-11 on brain microglia is uniquely human, because in nonhuman primates, Siglec-11 seems limited to peripheral tissue macrophages [81, 108, 109]. Moreover, microglia express a different Siglec-11 isoform than tissue macrophages [72, 110]. Microglial Siglec-11 lacks the exon encoding the innermost C2-set Ig domain of the extracellular protein part, but little is known about the functional consequences of this variation. When expressed as recombinant soluble IgG-Fc chimeras, the microglial isoform binds better to immobilized *E. coli* K1-derived polySia than the tissue macrophage form, and after transient expression in HEK293 cells, microglial Siglec-11 was secreted by ectodomain shedding as well as in exosomes [110]. The secreted Siglec-11, therefore, has the potential to bind to polySia on the surface of distant cells, but the relevance of this mechanism and its occurrence in vivo remain elusive.

As another feature of the human system, the inhibitory receptor Siglec-11 has a paired receptor, Siglec-16, which has a highly similar extracellular domain, but lacks the ITIM-bearing cytoplasmic tail [111–113]. Instead, Siglec-16 has a positively charged amino acid in its transmembrane region that interacts with the activating ITAM-containing adaptor DAP12 (Fig. 2). Putatively functional orthologs of human *SIGLEC16* have been detected by database comparisons in a number of other mammals, but not e.g. in rodents [114]. It seems however unique to the human population, that not all individuals have a functional *SIGLEC16* allele. The widespread occurrence of a *SIGLEC16P* pseudogene with a four base pair deletion, causing a reading frame shift, leads to an allelic frequency of 0.22 for the *SIGLEC16* allele and a penetrance for functional Siglec-16 of less than 40%, i.e. more than 60% of the human population are not able to produce functional Siglec-16 [80, 109, 111].

In its initial description, Siglec-16 was detected on tissue macrophages as well as on a rare population of cells in the human brain using antiserum raised against the short cytoplasmic tail of Siglec-16 [111]. More recently, newly established monoclonal antibodies against peptides from the extracellular domains of Siglec-11 and Siglec-16 enabled double immunofluorescence staining to demonstrate that the two paired Siglecs can occur together on human spleen macrophages [84]. As before [111], Siglec-16 was rarely detected by immunostaining in the brain, but database analysis indicated mRNA expression of Siglec-16 by microglia [84].

Concerning the assumed functions of Siglec-16, it has been suggested early on, that the switch from inhibitory to activating signaling in response to the same ligand may be an evolutionary strategy to balance responses to pathogens that interact with Siglec-11 and thereby exploit its inhibitory signaling to suppress the immune response of the host [111]. In this regard, it could be demonstrated that polySia encapsulated *E. coli* K1 binds equally well to Siglec-Fc chimeras comprising the first two Ig-domains of Siglec-11 or Siglec-16, whereas a sialic acid-deficient K1 strain lost the binding to Siglec-11 [84]. *E. coli* K1 also interacted with subpopulations of microglial cells transfected to express either Siglec-11 or Siglec-16, and after infection with *E. coli* K1 in vitro, more bacteria could be recovered from the Siglec-11 and less from the Siglec-16 transfected cells, as compared to controls transfected with empty vector [84]. However, in the Siglec-16 transfectants, the survival of sialic acid-deficient bacteria was also reduced, possibly due to a higher overall reactivity towards bacterial antigens. Similarly, and comparable to the effect of Siglec-E deficiency described above, a murine macrophage-like cell line transfected to express chimeric Siglec-E16, consisting of the extracellular part of Siglec-E fused to the transmembrane domain of Siglec-16, displayed higher inflammatory cytokine production in response to LPS stimulation [84]. Finally, mice were generated, in which Siglec-E was replaced by Siglec-E16. Similar to the inherent Siglec-E, expression of the chimeric receptor could be detected on blood neutrophils as well as on spleen and liver macrophages. One hour after intravenous injection of *E. coli* K1, significantly less bacteria were retrieved from blood, liver and spleen of homozygous Siglec-E16 transgenic as compared to Siglec-E wildtype mice. Concomitantly, blood levels of inflammatory cytokines were increased. Thus, replacing inhibitory Siglec-E by activating Siglec-16 signaling domains conveyed a protective innate immune response against a polySia-presenting pathogen [84]. It remains open if the altered responsiveness of the Siglec-E16 transgenic mice is caused by the loss of inhibitory Siglec-E, a gain of activating Siglec-16 signaling, or a combination of both.

Despite these implications of Siglec-16 to counteract a dampening of inflammatory responses by the polySia-Siglec-11 axis, its interplay with Siglec-11 under physiological conditions remains to be explored and virtually nothing is known about how the presence or absence of functional Siglec-16 affects neuroinflammation after insults or in neurodegenerative diseases, or how it may modulate the immune environment of polySia-positive brain tumors^1^. It is evident, though, that the Siglec-16 status has to be considered in translational studies concerning the polySia-mediated modulation of innate immune responses.

## PolySia interacts with the chemokine CCL21

Immature DCs in the periphery sense pathogens and capture antigens leading to their maturation and migration to lymphoid tissues, where they present the antigens to naïve T cells [115, 116]. After the initial discovery of polySia-NRP2 on the surface of human monocyte-derived DC [52], substantial work has been performed to unravel the role of polySia for chemotactic migration of DCs from the periphery to the lymph node in response to the chemokine CCL21 [reviewed in 21]. Despite clear evidence that polySia interacts with CCL21 and that polySia on DCs is required for CCL21-directed migration [117–119], a later study demonstrated that NRP2-negative DCs still respond to CCL21 and still carry polySia, leading to the identification of the CCL21 receptor CCR7 as a second polySia-presenting protein on the surface of DCs [120]. As shown in this study, polysialylation of CCR7 is indispensable for the chemotactic response during the interstitial migration of DCs before entering the lumen of lymphatic vessels. CCL21 carries a positively charged, polybasic C-terminal extension, which keeps the chemokine in an autoinhibited conformation. By binding to the C-terminal extension, polySia releases this autoinhibitory state and allows DCs to sense CCL21 gradients [120].

In microglia, CCR7 has been detected after activation with LPS or protein antigens and LPS-induced microglia exhibits CCR7-dependent chemotaxis towards CCL21 [121]. CCR7 was not included in the list of candidate polySia carriers in cultured microglia and cell-associated polySia could not be detected in LPS-induced microglia [50]. Unlike for dendritic cells, it therefore is highly improbable that polySia on the surface of LPS-induced microglia activates CCL21 for recognition by CCR7. However, as a rare example of a CC chemokine that binds to a CXC chemokine receptor, CCL21 can also bind to CXCR3 [122] and microglia obtained from CCR7 knockout mice are still able to respond to CCL21, whereas chemotactic migration towards CCL21 was abolished in microglia from mice that lack CXCR3 [123]. Thus, irrespective of activation state and of the receptor employed, microglia show chemotaxis towards CCL21. Importantly, CCL21 is exclusively released by damaged neurons, not glial cells, to activate microglia distant from a primary lesion [124]. In light of the strong binding interactions between polySia and CCL21 [120], it appears likely that polySia on proteins released by injury-activated microglia binds CCL21 from injured neurons. These interactions may modulate the perception and the chemotactic response of microglia towards CCL21 (Fig. 2).

## PolySia modulates release and toxicity of neutrophil extracellular traps

Another immunomodulatory function of polysialylated proteins released by microglia or OPCs may be their impact on neutrophil extracellular traps (NETs). NETs are extrusions of modified chromatin structures that are released by neutrophils as part of the first line of innate immune defense against pathogens [125], but show detrimental effects in TBI, stroke and other insults that involve leakage of the blood-brain barrier and inflammatory immune cell infiltration [4, 126, 127]. As shown in cell culture and in a mouse model of chronic obstructive pulmonary disease, polySia-NCAM accumulates in the trans-Golgi compartment of lung epithelial cells, and is released after stimulation with IL-1β or LPS by metalloproteinase-mediated ectodomain shedding [128, 129]. As shown in these studies, pre-incubation of NETs with soluble polySia or poySia-NCAM reduced NET-mediated cytotoxicity. In addition, polySia inhibits the release of NETs by interacting with lactoferrin, which forms shell-like structures around activated neutrophils [130], and interactions of polySia with histones and lactoferrin as NET components may impact on their composition and toxicity [131, 132]. It therefore will be important to analyze, if the release of polysialylated proteins, as predicted for TBI and related neuroinflammatory conditions, contributes to a better outcome by reducing the formation and cytotoxicity of NETs from infiltrating neutrophils in addition to a Siglec-dependent limitation of the neuroinflammatory response.

## Immunomodulation by polySia application in vivo

The immunomodulatory potential of polySia application in vivo was explored in transgenic mice expressing Siglec-11 in mononuclear phagocytes. In these mice, intravitreal injection of polySia with avDP20 reduced laser-induced vascular damage in the retina as a model of age-related macular degeneration, and inhibited microglia and macrophage activation [87]. With a higher dose of polySia avDP20, both effects were also detected in wildtype mice. This has been attributed to a lower sensitivity of murine Siglec-E, determined by comparing the inhibitory effects of polySia avDP20 on LPS-induced activation of mouse ES cell-derived microglia and human THP1 macrophages. As discussed above (*Immunomodulation by the polySia-Siglec axis*), the difference in vitro may actually be due to different polySia chain lengths required for the activation of Siglec-E and Siglec-11. The same may apply to the higher sensitivity of the Siglec-11 transgenic mice towards the polySia-induced reduction of retinal damage, but, in addition or instead, the transgenic expression of Siglec-11 on top of endogenous Siglec-E may augment sensitivity by raising the density of polySia-responsive inhibitory Siglecs. Nevertheless, the study by Karlstetter et al. [87] provides proof-of-principle for the therapeutic potential of polySia applications.

Another finding of this study was a reduced deposition of the complement membrane attack complex in the laser-induced retina lesions after polySia application, which was observed in Siglec-11 transgenic and in wildtpye mice. In vitro, LPS-induced deposition of complement was reduced by polySia avDP20 indicating an inhibition of the alternative complement pathway [87]. This was followed up in a recent study showing that polySia avDP20 directly interacts with properdin, a positive regulator of the alternative pathway, and reduces properdin-mediated complement deposition [133]. Properdin is an oligomeric protein with an unusually high isoelectric point (> pH 9.5). Under physiological conditions, therefore, properdin is highly positively charged, and tends to bind to polyanionic structures such as sulfated glycosaminoglycans on the cell surface or in the extracellular environment [134, 135]. This may be the reason, why polyanionic polySia interacts with properdin and why soluble forms of polySia are able to compete with complement activation by properdin at the surface of lesioned cells [133].

In these in vitro experiments, a significant inhibition of properdin binding to lesioned cells by polySia avDP20 was observed at concentrations between 32 and 45 µM. In contrast, 1.3 µM of polySia avDP20 and 30 nM of avDP50 were sufficient for the Siglec-E dependent inhibition of LPS-induced murine microglia [60, 87], pointing towards a lower threshold for activation of the polySia-Siglec axis as compared to polySia-properdin interactions. Interestingly, the release of inflammatory cytokines from microglia as observed in brain injury and neurodegenerative disorders induces astrocyte activation towards a complement-producing neurotoxic phenotype [136]. Considering this microglia-astrocyte crosstalk in the initiation of complement-mediated neurotoxicity, it will be an important direction of future research to explore if an impact of polySia on complement deposition in injured neural tissue is due to inhibitory interactions between polySia and properdin, or based on a Siglec-dependent inhibition of microglia leading to reduced complement production by reactive astrocytes, or possibly a combination of both.

Activation of microglia is prominent in the substantia nigra of patients with Parkinson’s disease, and observed prior to the loss of dopaminergic neurons, the neuropathological hallmark of parkinsonism. This implicates that microglia mediates detrimental neurotoxic effects, but such a causal link is disputed and a contribution of peripheral inflammation is discussed [137]. In mice, systemic challenge by intraperitoneal injections of LPS leads to inflammatory activation of microglia and to a complement-dependent loss of dopaminergic neurons in the substantia nigra [138]. In the model of Siglec-11 transgenic mice, effects of systemic LPS administration on the whole-brain transcriptional signature of inflammatory microglia activation could be ameliorated by concomitant intraperitoneal injections of polySia avDP20 [139]. Similar to the previous study on retina damage [87], and with the same caveats discussed above, polySia had no such effect in LPS treated wildtype mice. Likewise, effects of polySia on the LPS-induced expression of genes related to the complement pathway were also restricted to the Siglec-11 transgenic mice [139]. This seems to contradict the polySia effect on the formation of the complement-mediated membrane attack complex in the lesioned retina [87], but may be explained by different sensitivities in the detection of transcriptional and immunohistological changes.

Furthermore, in both wildtype and Siglec-11 transgenic mice, systemic polySia treatment reduced the LPS-induced increase of the microglial markers Iba-1 and CD68 detected by immunostaining and also prevented the loss of dopaminergic neurons in the substantia nigra [139]. Again, the apparent discrepancies between the effects of polySia avDP20 on global transcriptional changes of brain microglia and localized effects detected by immunohistochemistry in the substantia nigra might be due to different detection limits. Nonetheless, it is evident that polySia treatment has potent anti-inflammatory and neuroprotective effects also in wildtype mice, most probably mediated by interactions with Siglec-E. It should, however, be noted that systemic LPS administration may trigger neuroinflammation and neurodegeneration by an initial, temporary increase of TNF production in the periphery, because intraperitoneal TNF injections reproduced the effects of LPS, and in TNF receptor deficient mice, LPS injections were unable to induce any of the pro-inflammatory changes observed in the brain of wildtype mice [140]. It therefore seems possible that intraperitoneal polySia application exerts its effect not by acting on brain microglia after crossing the blood-brain-barrier, but by inhibition of peripheral inflammation and TNF production, for example in Kupffer cells, the liver-resident tissue macrophages, which are the major source of circulating TNF and known to express Siglec-E in mice and Siglec-11 in humans [80, 141].

## Perspectives

An important task for upcoming research will be to establish if and to what extent and under which neuropathological conditions protein-bound polySia can be released by activated microglia or possibly OPCs in vivo. Up to know, we only have first hints from in vitro studies on continuous shedding of polysialylated proteins by LPS-induced microglia in combination with the immunohistochemical localization of intracellular polySia in a transient activation state of injury-induced microglia in situ. Sensitive methods for the detection of soluble polySia-ESL-1, polySia-NRP2, or even polySia-SynCAM 1 in extracts from brain tissue or in cerebrospinal fluid will allow to determine if shedding of polysialylated proteins by activated microglia, invading monocyte-derived macrophages, and possibly OPCs occurs during acute or chronic neuroinflammation after TBI or other brain insults, in neurogenerative diseases, or in the immune environment of brain tumors. Once established, a major challenge will be to explore the consequences of polySia release in rodent models and in human diseases involving neuroinflammation.

As already discussed in the respective sections above, it also seems essential to analyze if polySia affects complement deposition via direct or indirect mechanisms in vivo. Further unexplored areas are the possible interactions of soluble forms of polySia with the chemokine CCL21 and their potential to modulate microglial chemotaxis, as well as the possibility that polySia interactions with NETs may reduce neurotoxic consequences of invading neutrophils after brain insults. Concerning translational studies on the role of Siglecs as polySia receptors, it will be critical to define commonalities and differences in polySia interactions, activation, and trafficking between murine Siglec-E and human Siglec-11 or Siglec-16. Even more crucial, though, will be to establish valid systems to study the physiological role of Siglec-16 and its engagement as potentially activating counterpart of Siglec-11 in responses to polySia released under neuroinflammatory conditions, or presented, e.g., on the surface of tumor cells^1^.

An interesting direction of future research could also be to investigate the functional consequences of a single-nucleotide polymorphism (SNP) in *SIGLEC11*, which has been associated with Alzheimer’s disease (AD) in a recent genome wide association study (GWAS) meta-analysis [142]. Associations of several other microglial genes were detected in this study, and a pathway enrichment analysis confirmed an involvement of pathways related to innate immunity and microglial activation in Alzheimer’s disease, as shown by a number of previous studies. Interestingly, genetic variants of two other microglial receptors, the ITAM linked activating TREM2 and the ITIM containing inhibitory Siglec-3 (CD33) have been firmly linked to an increased risk for developing AD [143, 144]. Together with mounting evidence for the immunomodulatory and therapeutic potential of polySia, this warrants further studies on the polySia-Siglec axis in AD and other neurodegenerative conditions.

To conclude, the polySia-Siglec axis emerges as a promising immune checkpoint and target for intervention with a largely unexplored impact on immune balance and neuroinflammation in the diseased brain.

## Data Availability

Data sharing not applicable to this article as no datasets were generated or analyzed during the current study.

## Footnotes

1 During peer reviewing of this manuscript a study was published showing that the polySia-Siglec-16 axis has a proinflammatory impact on tumor-associated macrophages (TAM) in glioblastoma. This was linked to increased survival of glioblastoma patients that were *SIGLEC16* carriers and had tumors presenting with high levels of polySia-NCAM on the tumor cells (Thiesler et al., Proinflammatory macrophage activation by the polysialic acid-Siglec-16 axis is linked to increased survival of patients with glioblastoma. Clin. Cancer Res. 10.1158/1078-0432.CCR-22-1488. Online ahead of print, 14.04.2023).
